# Dosimetric and motion analysis of margin-intensive therapy by stereotactic ablative radiotherapy for resectable pancreatic cancer

**DOI:** 10.1186/1748-717X-6-146

**Published:** 2011-10-28

**Authors:** John H Heinzerling, Ross Bland, John C Mansour, Roderich E Schwarz, Ezequiel Ramirez, Chuxiong Ding, Ramzi Abdulrahman, Thomas P Boike, Timothy Solberg, Robert D Timmerman, Jeffrey J Meyer

**Affiliations:** 1 Department of Radiation Oncology, University of Texas Southwestern Medical Center, Dallas, TX, USA

**Keywords:** stereotactic, pancreatic cancer, abdominal compression

## Abstract

**Background:**

The retroperitoneal margin is a common site of positive surgical margins in patients with resectable pancreatic cancer. Preoperative margin-intensive therapy (MIT) involves delivery of a single high dose of ablative radiotherapy (30 Gy) focused on this surgically inaccessible margin, utilizing stereotactic techniques in an effort to reduce local failure following surgery. In this study, we investigated the motion of regional organs at risk (OAR) utilizing 4DCT, evaluated the dosimetric effects of abdominal compression (AC) to reduce regional motion, and compared various planning techniques to optimize MIT.

**Methods:**

10 patients were evaluated with 4DCT scans. All 10 patients had scans using AC and seven of the 10 patients had scans both with and without AC. The peak respiratory abdominal organ and major vessel centroid excursion was measured. A "sub-GTV" region was defined by a radiation oncologist and surgical oncologist encompassing the retroperitoneal margin typically lateral and posterior to the superior mesenteric artery (SMA), and a 3-5 mm margin was added to constitute the PTV. Identical 3D non-coplanar SABR (3DSABR) plans were designed for the average compression and non-compression scans. Compression scans were planned with 3DSABR, coplanar IMRT (IMRT), and Cyberknife (CK) planning techniques. Dose volume analysis was undertaken for various endpoints, comparing OAR doses with and without AC and for different planning methods.

**Results:**

The mean PTV size was 20.2 cm^3^. Regional vessel motion of the SMA, celiac trunk, and renal vessels was small (< 5 mm) and not significantly impacted by AC. Mean pancreatic motion was > 5 mm, so AC has been used in all patients enrolled thus far. AC did not significantly increase OAR dose including the stomach and traverse colon. There were several statistically significant differences in the doses to OARs as a function of the type of planning modality used.

**Conclusions:**

AC does not significantly reduce the limited motion of structures in close proximity to the MIT target and does not significantly increase the dose to OARs that can be displaced by the compression plate. The treatment planning techniques evaluated in this study have different advantages with no clearly superior method in our analysis. Dose to adjacent vessels may be reduced with 3DSABR or IMRT techniques, while conformality is increased with IMRT or CK.

## Background

An estimated 43,000 people in the United States were diagnosed with pancreatic ductal adenocarcinoma (PA) in 2010 [1]. PA is the fourth leading cause of cancer-related death in the US, with over 36,000 deaths in 2010.^1 ^The majority of patients with PA present with metastatic disease at the time of diagnosis with a median survival of 3-6 months [2]. While approximately 20-25% of patients are diagnosed with localized disease that is amenable to surgical resection,[2] even these patients that undergo surgical resection have a 5 year survival rate of less than 20% [3]. Following surgical resection of PA originating in the head of the gland, at least 20% of patients will have positive margins, typically located in the retroperitoneal space medial and posterior to the pancreas, and 65% of patients will have involved lymph nodes [4]. Positive surgical margins in these patients is a poor prognostic factor and associated with decreased survival [5]. Following surgical resection, local failure can occur in 50-80% of patients, with the retroperitoneal margin as the most common location of isolated local failure [6-11].

Because of the high incidence of local failure in surgically resected PA, radiation therapy has been investigated both preoperatively and postoperatively as adjuvant therapy to help reduce this risk in patients with potentially curable disease [12-18]. Some of these trials showed a benefit to adjuvant radiation therapy following surgical resection, while others showed a detriment or no benefit, leading to controversy surrounding the role of radiation therapy in addition to chemotherapy for patients with surgically resected disease. Patients with early dissemination of disease would not benefit from additional local therapy such as radiation, making it difficult within this patient population to show a survival advantage to radiation even if local control is improved. In contrast to radiation therapy, important trials have demonstrated a clear survival benefit to adjuvant chemotherapy following surgical resection, leading to standardization of chemotherapy as adjunctive therapy in these patients [19]. Thus, current ideal treatment of patients with resectable PA includes complete surgical resection with early delivery of adjuvant chemotherapy to address systemic disease.

New radiation techniques may be exploited to allow the local control benefits of radiation therapy to be administered without significantly delaying systemic chemotherapy or causing decline related to toxicity. Stereotactic ablative radiotherapy (SABR), also known as stereotactic body radiation therapy (SBRT), utilizes advanced techniques of immobilization, image guidance, and unique field arrangements to deliver precise, oligofractionated radiotherapy to a variety of tumor types. SABR has been established as a technologically innovative therapy for early stage non-small cell lung cancer (NSCLC) and has emerged as the standard treatment option for medically inoperable patients through prospective, multi-institutional trials [20,21]. SABR for locally advanced, unresectable pancreatic cancer has been investigated in both phase I and II trials [22-24]. In these trials, doses ranging from 15-45 Gy in 1-3 fractions were administered to the primary tumor in patients with locally advanced, unresectable PA. In one phase II trial, 45 Gy in 3 fractions was used to treat the entire primary, unresectable tumor plus margin, but associated toxicity was high in this group of 22 patients [24]. Increased levels of grade 2 and greater nausea and pain were seen in a significant proportion of treated patients, leading to decline in performance status, and increased analgesic use. In four patients, severe mucositis or ulceration of the stomach or duodenum was observed on post-treatment endoscopy. Another phase I/II experience had more encouraging results for use of SABR in locally advanced PA when treated with smaller margins again in unresectable disease [22,23]. The phase I portion of the trial escalated single doses of radiation from 15 to 25 Gy delivered with the Cyberknife^® ^radiosurgery system with no grade 3 or higher toxicity observed. The phase II portion of the trial treated patients with fractionated IMRT and 5-FU chemotherapy prior to a SABR boost of 25 Gy based on the phase I experience. Two out of 16 patients experienced grade 3 toxicity consisting of gastroparesis. Again, duodenal ulcers were seen 4-6 months after treatments, though all were managed medically. Median survival was 33 weeks for the 16 patients. This trial demonstrated the feasibility of utilizing SABR as a boost for locally advanced disease, and although local control rates were promising for this limited experience, overall survival did not seem to improve over expected rates observed with conventional chemoradiation. Each of these trials studied patients with larger, unresectable tumors, with significant irradiated volumes required to treat the entire tumor plus margin. As a consequence, relatively radiosensitive normal organs at risk (OAR) such as the duodenum, distal stomach, and remaining small bowel limited tolerance of SABR.

We define Margin Intensive Therapy (MIT) as a new concept that delivers ablative doses of radiation only to the tumor border around unresectable structures such as the superior mesenteric artery (SMA) and retroperitoneal margin preoperatively with the goal of reducing the positive margin rate and, hence, local failure rate in this area. As opposed to conventional radiation for PA which includes a the entire tumor and regional lymph nodes in the treatment volume, MIT focuses an single ablative dose (e.g., 30 Gy) of radiation to a very small at risk area in contrast to traditional delivery of 50.4 Gy in 28 fractions to the pancreatic tumor and regional lymph nodes. MIT complements surgery as it treats the area most difficult for surgeons to resect and obtain clear margins. To deliver MIT, stereotactic techniques are used, but unlike the previous trials exploring SABR for locally advanced PA, MIT delivers dose away from sensitive structures such as the duodenum that have led to high toxicity levels in previous trials. By delivering the dose in a single fraction, surgical resection will not be delayed in these patients, who can go on to receive adjuvant systemic therapy without delay after recovery.

To explore the concept of MIT, we have initiated a phase I trial at our institution evaluating the safety and toxicity of preoperative MIT followed by surgical resection with pancreaticoduodenectomy in patients with newly diagnosed, resectable PA. This is novel use of SABR in its targeting of a high risk subvolume in operable patients rather than the entire tumor in unresectable patients. Additionally, the respiratory associated motion of structures in close proximity to the target volume for PA has not been thoroughly explored. The effect of abdominal compression as a tool to reduce respiratory tumor motion, as is used at our institution,[25] on unique adjacent structures such as the SMA and renal vessels is also unknown. Because abdominal compression may displace OARs such as the transverse colon, stomach, and small intestine towards the target, there may be an effect on dose to these structures when abdominal compression is utilized in MIT. Finally, since many planning techniques can be utilized to deliver MIT with SABR methods, we performed a planning comparison for this novel concept in an attempt to optimize MIT.

## Methods

### Patient selection

For these comparisons, we chose six patients who previously had 4D CT scans taken with and without abdominal compression as previously described [25]. These patients did not have PA; most were treated with SABR for lung primary or metastatic tumors. However, since the target volume was based on the vessel location and the retroperitoneal margin of the pancreatic head, PA was not needed for volume delineation in this study. In addition to these 6 patients, the first 4 patients enrolled on the phase I trial then underwent 4D CT scans. This is an ongoing clinical trial that has been approved by the Institutional Review Board in compliance with the Helsinki Declaration. In total 7 patients had 4DCT scans both with and without AC, and 3 patients had scans with AC only (total 10 patients). Patients eligible for the trial had pathologically confirmed PDAC with surgically resectable disease as determined by a surgical oncologist at our institution based on CT or MRI of the abdomen.

### 4D-CT Scan Acquisition and Reconstruction

Patients were simulated in an Elekta Stereotactic Body Frame^® ^as described previously [26]. All 4D scans were acquired with the Philips Brilliance 190P multi-slice 4D-CT scanner (Philips Medical Systems, Bothell, WA). The respiratory cycle signal was acquired using the Bellows System (Philips Medical Systems, Bothell, WA). This system uses an elastic strap that was attached to the abdominal or thoracic region and correlates chest wall motion with the respiratory cycle. Phase sorting was performed and images from the 10 phases were automatically reconstructed by the Philips software. The phases were defined as 0% to 100% corresponding to the point in the respiratory cycle which the images represented with 0% representing peak inspiration. In addition, the peak expiratory phase was reconstructed based on the respiration cycle data analyzed by the Philips software. All phases were evaluated within the Philips CT viewing system to ensure that the maximum diaphragmatic and tumor movement were represented by the peak inspiratory and expiratory phases. This procedure was repeated consistently for both the AC and non-compression (NC) 4D CT scans.

### Evaluation of tumor and organ motion

The CT series representing peak inspiration and peak expiration were transferred to the Philips Pinnacle v8.0 m ^® ^treatment planning system for viewing. Image fusion was used to ensure correlation between the two CT series. Automatic image fusion was performed initially and evaluated using manual fusion of the frame fiducials. Within the peak inspiration CT series, the kidneys, pancreas, liver, spleen, SMA, renal vessels, liver, stomach, and duodenum were contoured. These contours were then transferred onto the peak expiratory phase and adjusted to fit the position of these same structures at that point of the respiratory cycle. A point of interest representing the centroid point of that particular structure's contour was then generated for each structure in both respiratory phases. The position of the centroid points were then compared to determine the peak movement of each structure in the SI, lateral, and anterior-posterior (AP) directions. Overall movement was calculated as the magnitude of the vector of the three directions. Motion in all three directions along with overall vector motion was compared with the AC and NC scans utilizing a two-tailed paired t-test with significant differences defined as p < 0.05.

### Evaluation of Dosimetric Effect of Abdominal Compression on OARs

**Figure 1:**
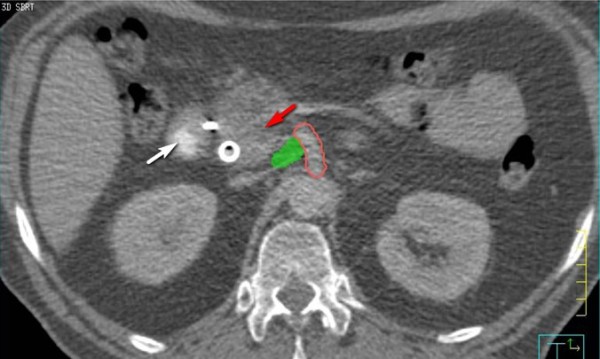
**"Sub-GTV" volume is shown in green**. Small volume lateral and posterior of the pancreatic head tumor (red arrow). Volume is significant distance from sensitive structures such as duodenum (white arrow).

Average intensity projection CT scans were reconstructed from both the AC and NC 4D CT scans for the 10 patients and imported into Pinnacle v8.0 m. Seven of the ten patients had NC 4D CT scans available for analysis and all 10 patients had AC 4D CT scans available. All patients were scanned supine with arms above their head utilizing IV contrast. The "sub-GTV" (Figure 1) target volume was defined by the radiation oncologist and surgical oncologist consisting of a small volume typically posterior and lateral to the SMA that included the projected surgical margin of resection and any retroperitoneal or peripancreatic nodal tissue in the area of the tumor but outside the anticipated feasible resection field. This volume was then expanded in all directions by 3 mm in the axial plane and 5 mm in the superior-inferior direction to constitute the PTV. Organs at risk including the liver; stomach; duodenum; small bowel; ascending, descending, and traverse colon; SMA, renal vessels, celiac trunk, kidneys, and spinal cord were identified and contoured on both scans. Identical 3D conformal SABR (3DSABR) plans were then constructed for both AC and NC studies. These plans consisted of 10 non-coplanar beams with conformal blocking to the PTV. We typically employ a negative margin blocking scheme to create an isotropic dose falloff around the target [26]. The prescription dose was 30 Gy in 1 fraction as was planned in the phase 1 trial, and plans were prescribed to the 65-85% isodose line to ensure that 95% of the PTV volume was covered by 100% of the prescription dose and that 99% of the PTV volume was covered by 90% of the prescription dose. Doses to the contoured OARs were then compared for the AC and NC plans utilizing a two-tailed paired t-test. Differences were considered significant for p < 0.05.

### Comparison of Dosimetric Outcomes for Three Different Planning Methods

Average intensity projection AC scans were then planned utilizing three different planning techniques. The first planning technique was 3DSABR is described above. Additionally, a coplanar IMRT (IMRT) plan was generated in Pinnacle utilizing 13 coplanar beams directed at the PT was generated. Objectives were created for the duodenum, liver, small bowel, and spinal cord to meet institutional constraints for single-fraction SABR to 30 Gy in 1 fraction (Table 1). Plans were prescribed based on coverage parameters identical to those of 3DSABR. Finally, Cyberknife^® ^(CK) plans were generated for all 10 patients after exporting average scans and contours to the MultiPlan^® ^treatment planning system v. 4.0 (Accuray, Sunnyvale, CA). Plans were generated utilizing the sequential optimization function typically with 2 collimators based on PTV size (typically 15 mm and 25 mm). Objectives were defined based on OAR constraints and PTV conformality as described above for IMRT planning. Plans were again assessed based on the coverage parameters above, typically to the 60-70% isodose line.

**Table 1:** OAR dose constraints used for planning for the phase I trial

Critical Structure Dose Limits				
Serial Tissue	Volume	Volume Max (Gy)	Max Point Dose (Gy)	Endpoint (≥ Grade 3)
Spinal Cord and medulla	< 0.35 cc	10 Gy	14 Gy	myelitis
	< 1.2 cc	7 Gy		
Spinal Cord Subvolume (5-6 mm above and below level treated)	< 10% of subvolume	10 Gy	14 Gy	myelitis
Skin	< 10 cc	23 Gy	26 Gy	ulceration
Stomach	< 10 cc	11.2 Gy	12.4 Gy	ulceration/fistula
Duodenum*	< 5 cc < 10 cc	11.2 Gy 9 Gy	12.4 Gy	ulceration
Jejunum/Ileum*	< 5 cc	11.9 Gy	15.4 Gy	enteritis/obstruction
Colon*	< 20 cc	14.3 Gy	18.4 Gy	colitis/fistula
Liver	700 cc	9.1 Gy		Basic Liver Function
Renal cortex (Right & Left)	200 cc	8.4 Gy		Basic renal function

### Dosimetric analysis and statistical methods

Dose volume results for 3DSABR, IMRT, and CK plans for each patient were recorded and included maximum, minimum, and mean dose for the contoured OARs. Specific dose-volume relationships including dose to 5 cc of the duodenum, 5 cc of the non-duodenum small intestine, 10 cc of the stomach, 100 cc of the bilateral kidneys, 700 cc of the liver, 20 cc of the colon, and 0.3 cc of the spinal cord were recorded and compared. A conformality index (CI) was calculated for each planning technique by calculating the ratio of the volume receiving the prescription dose (30 Gy) to the PTV volume. A two tailed paired t test was used to compare doses for parameters above for each of the three planning methods. Differences were considered significant for p < 0.01667 based on a Bonferroni correction for level of the p-value significance.

## Results

### Patient characteristics

Thus far, 4 patients have been enrolled on the phase I trial and each received 30 Gy in 1 fraction to the PTV followed by surgical resection. All patients met eligibility criteria as described above. The primary endpoints of the trial include preoperative toxicity and postoperative surgical morbidity. As described above, 6 other patients were included in this analysis not enrolled on the phase I trial that had previous 4D CT scans with and without AC for analysis of motion and planning techniques. The mean sub-GTV size for the 10 patients was 4.4 cm^3 ^(3.4-6.6 cm^3^) and the mean PTV size was 20.2 cm^3 ^(15.6-24.9 cm^3^).

### Motion Analysis

For each organ, mean motion values for all three directions and vector motion with and without AC are shown in Table 2. Motion of structures adjacent to the PTV, including the SMA and renal vessels had very little respiratory-associated motion. For the trial, AC was used in all patients based on motion of fiducial markers place in the head of the pancreas with motion evaluated with fluoroscopy. AC did not significantly affect motion of most organs, but did significantly reduce AP motion of the stomach (p = 0.009), AP motion of the pancreas (p = 0.009), and vector motion of the SMA (p = 0.02).

**Table 2:** Mean motion and ranges in cm for regional structures without (top) and with (bottom) abdominal compression

**Mean Motion without compression (cm)**								
Organ	AP	Range	Lat	Range	SI	Range	Vect	Range
Liver	0.07	0.02-0.17	0.41	0.16-0.53	0.82	0.42-1.74	0.95	0.50-1.82
Spleen	0.18	0.14-0.30	0.32	0.26-0.68	0.70	0.11-1.87	0.84	0.37-2.00
Stomach	0.19	0.01-0.42	0.26	0.15-0.46	0.60	0.4-0.99	0.76	0.62-1.01
Duodenum	0.20	0.02-0.27	0.25	0.03-0.68	0.49	0.06-1.02	0.58	0.18-1.26
Pancreas	0.29	0.02-0.26	0.24	0.10-0.38	0.52	0.12-0.89	0.68	0.32-0.98
Celiac trunk	0.14	0.08-0.27	0.23	0.04-0.42	0.28	0.07-0.75	0.39	0.22-0.90
SMA	0.13	0.06-0.46	0.21	0.04-0.48	0.24	0.07-0.45	0.42	0.39-0.65
R kidney	0.08	0.01-0.08	0.28	0.03-0.55	0.64	0.19-0.82	0.60	0.19-0.99
L kidney	0.06	0-0.14	0.09	0-0.28	0.32	0.11-0.74	0.37	0.16-0.80
R renal vessels	0.21	0.05-0.42	0.33	0.36-0.41	0.54	0.31-1.32	0.70	0.58-1.39
L renal vessels	0.29	0.03-0.33	0.12	0.03-0.26	0.38	0.21-0.93	0.57	0.24-1.02
Mean motion with compression (cm)								
Organ	AP	Range	Lat	Range	SI	Range	Vect	Range
Liver	0.11	0.08-0.19	0.23	0.03-0.66	0.48	0.2-0.68	0.56	0.25-0.97
Spleen	0.19	0.01-0.32	0.22	0.08-0.67	0.63	0.06-1.25	0.71	0.10-1.45
Stomach	0.16	0-0.28	0.11	0.05-0.23	0.36	0.08-0.46	0.46	0.14-0.50
Duodenum	0.10	0.02-0.19	0.09	0.01-0.27	0.36	0.06-0.58	0.40	0.07-0.61
Pancreas	0.28	0.02-0.89	0.08	0.01-0.1	0.40	0.08-0.57	0.54	0.13-0.90
Celiac trunk	0.13	0-0.49	0.12	0.01-0.25	0.23	0-0.30	0.32	0.01-0.60
SMA	0.11	0.04-0.17	0.09	0.01-0.17	0.15	0-0.27	0.24	0.10-0.28
R kidney	0.05	0.04-0.21	0.19	0.12-0.48	0.52	0.08-1.59	0.56	0.17-1.67
L kidney	0.05	0.01-0.07	0.12	0-0.12	0.45	0.09-0.92	0.48	0.12-0.93
R renal vessels	0.15	0.03-0.31	0.22	0.03-0.43	0.46	0.23-0.87	0.56	0.41-0.89
L renal vessels	0.24	0.01-1.15	0.18	0.06-0.36	0.33	0.04-0.54	0.52	0.16-1.28

### Dosimetric Effect of Abdominal Compression

Mean doses to the contoured OARs for the same 3DSABR plan for patients with and without AC are shown in Table 3. AC did not significantly increase minimum, mean, or maximum doses to any of the OARs including the stomach or transverse colon which can be displaced towards the target by the AC plate.

**Table 3:** Doses in cGy for regional OAR for 3DSBRT plans on simulation scans with and without AC

Organ	Mean dose without AC (cGy)	Mean dose with AC (cGy)	p value
L kidney	196.8	172.7	0.48
L renal vessels	701.8	629.8	0.70
R kidney	182.3	218.3	0.36
R renal vessels	397.9	460.6	0.60
Ascending colon	135.2	141.4	0.86
Celiac trunk	2459.0	2214.9	0.49
Spinal cord	233.4	248.7	0.76
Descending colon	121.9	133.2	0.78
Duodenum	484.8	531.0	0.48
Liver	132.9	108.7	0.27
SMA	2818.4	2951.8	0.77
Small bowel	235.2	238.3	0.95
Stomach	159.9	121.8	0.17
Transverse colon	192.9	197.8	0.90

### Comparative dosimetric analysis

**Figure 2:**
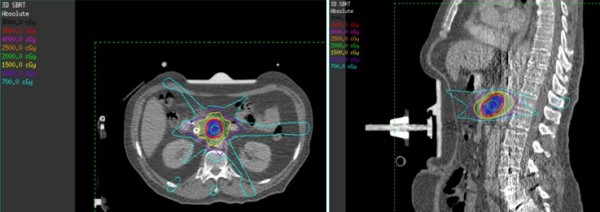
**Example of 3DSABR plan in axial and sagittal planes**.

**Figure 3:**
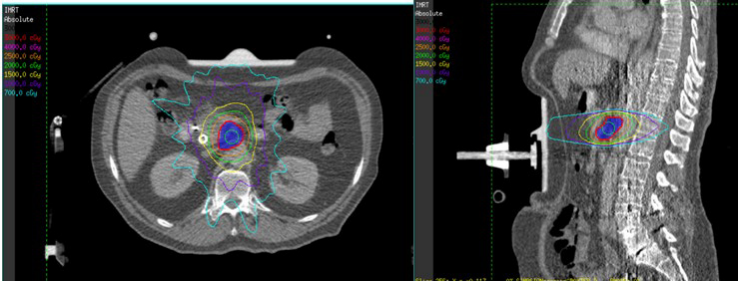
**Example of coplanar IMRT plan in axial and sagittal planes**.

**Figure 4:**
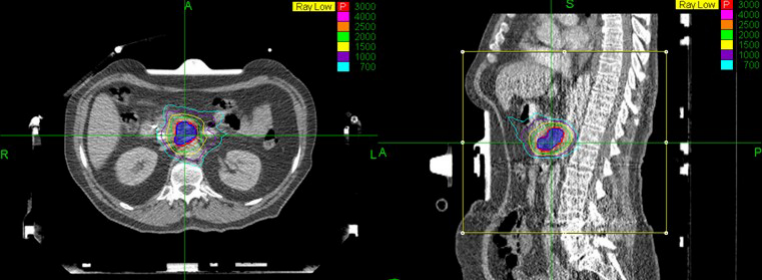
**Example of Cyberknife plan in axial and sagittal planes**.

A total of thirty plans were generated for this comparison, including 10 3DSABR, 10 IMRT, and 10 CK plans and were evaluated for CI and OAR dose volume criteria of interest. All plans met the basic dosimetric constraints used for this trial (Table 1). Figures 2, 3, and 4 show examples of 3DSABR, IMRT, and CK plans for the same patient for the described PTV volume. Results for significant differences between various dosimetric parameters are illustrated in Table 4. 3DSABR was superior to IMRT for the mean dose to the left renal vessels (6.3 vs. 9.5 Gy) (p = < 0.001), and mean (4.6 vs. 9.1 Gy) (p = 0.004) and maximum (15.1 vs. 20.6 Gy) (p = 0.003) doses to the right renal vessels. 3DSABR was superior to CK for the mean dose to the left renal vessels (6.3 vs. 8.3 Gy) (p = 0.002). CK was superior to IMRT for dose to 20 cc of the transverse colon (4.1 vs. 7.7 Gy) (p = 0.003). CK was superior to 3D SABR with respect to the target conformality index (CI) (1.19 vs. 1.29) (p = 0.008) and the dose to 20 cc of the transverse colon (4.1 vs. 5.8 Gy) (p = 0.003). IMRT was superior to 3D SABR for the mean dose to the liver (82 vs. 109 cGy) (p = < 0.001), max dose (30.7 vs. 36.0 Gy) (p = < 0.001) and mean dose (27.4 vs. 29.5 Gy) (p = 0.015) to the SMA, and CI (1.15 vs. 1.29) (p = 0.001). IMRT was superior to CK for the maximum dose to the celiac trunk (29.6 vs. 33.7 Gy) (p = 0.007), and the maximum (30.6 Gy vs. 35.7 Gy) (p = 0.001) dose to the SMA. Other reported significant outcomes are shown in Table 4.

**Table 4:** Significant dosimetric outcomes observed when comparing 3DSBRT, IMRT, and CK planning techniques

Organ	Parameter	Dose 3DSBRT (cGy)	Range (cGy)	Dose CK (cGy)	Range (cGy)	Dose MRT (cGy)	Range (cGy)	p value	Superior Modality
L kidney	Mean dose	172.7	80-294	148.0	86-249.8	297.3	183.4-410.4	< 0.001	3DSBRT and CK
L renal vessels	Mean dose	629.8	101-1431	828.0	178-1585	952.5	104.3-1621.7	< 0.001	3DSBRT
R kidney	Mean dose	109.1	50-220	81.5	41-150	217.7	66-429	0.007	3DSBRT and CK
R renal vessels	Maximum dose	1508.5	764-2503	1306.7	310-2566	2063.5	981-2984	0.001	3DSBRT and CK
R renal vessels	Mean dose	460.6	130-858	622.3	87-2380	909.2	253-1878	0.003	3DSBRT
Celiac trunk	Maximum dose	3284.9	1954-3754	3371.9	2880-3904	2958.8	2681-3286	0.007	IMRT
Duodenum	Mean dose	509.5	305-745	276.6	106-543	371.2	230-717	< 0.001	CK and IMRT
Duodenum	Dose to 5 cc	1108.6	840-1310	779.7	380-1273	845.9	598-1047	0.010	CK and IMRT
Liver	Mean dose	108.7	67-178	111.7	42-218	82.1	37-128	< 0.001	IMRT
SMA	Maximum dose	3551.0	2754-3815	3572.6	1618-4147	3068.0	1861-3373	0.001	IMRT
SMA	Mean dose	2951.8	913-3619	2999.4	859-3905	2745.7	533-3123	0.015	IMRT
Transverse colon	Dose to 20 cc	582.9	203-916	408.5	0-844	769.5	454-1134	0.003	CK
**Dosimetric value**	**Definition**	**Ratio value**	**Range**	**Ratio value**	**Range**	**Ratio value**	**Range**	**p value**	**Superior Modality**
Conformality index	Ratio	1.29	1.16-1.50	1.19	1.14-1.23	1.15	1.06-1.18	< 0.001	CK and IMRT

## Discussion

PA continues to be a leading cause of cancer related death in the U.S. with the majority of patients presenting with metastatic, incurable disease [1,2]. Despite improved imaging techniques utilized preoperatively to assess the likelihood of achieving a margin-negative surgical resection, about 20% or more of patients will have positive margins after pancreatoduodencetomy, with many more having "close" (1-2 mm) margins [4,5]. The main margin of interest for pancreatic head tumors is the retroperitoneal surgical margin, defined here as the tissue adjacent to the proximal SMA [27]. This space is invested with lymphatics and a rich plexus of nerves, and pancreatic tumor cells can infiltrate the perineural spaces making complete surgical excision impossible without excision of the artery itself in many cases [28]. Autopsy and clinical series have demonstrated high rates of residual tumor around the SMA and that these areas can grow as true local recurrences visible on CT [6-8,29]. Using a definition that tumor within 1 mm of the inked surgical specimen constituted a 'positive margin' Esposito et al showed that 76% of patients undergoing resection for PA had an R1 surgery [30]. Using the same criteria, Verbeke et al found the posterior resection margin to be involved in more than 50% of cases [31]. Our 'sub-GTV' was designed to encompass this high-risk soft tissue volume for pancreatic head tumors.

Given the high rates of close and positive margins and the attendant high rates of local failure, adjuvant fractionated radiation, typically delivered concurrently with chemotherapy, is commonly employed in the treatment of resected pancreatic cancer. Despite evidence that radiation does reduce local failure after surgical resection, adjuvant radiation has associated toxicities and can delay initiation of "full-dose"systemic therapy [9]. Moreover, positive surgical margins in the retroperitoneal area may portend aggressive disease biology that will minimize the chance that local therapy will improve survival. "Clearing" the retroperitoneal margin can be accomplished with resection of the SMA itself, but this has not, in general, been associated with high rates of long-term disease-free survival [32]. In other words, patients may indeed experience local failure, but typically in the setting of distant disease failure as well, with the latter ultimately leading to patient death. As systemic therapies continue to improve, however, the value of local control will likely increase. In addition, patients with close (< 2 mm) but technically negative surgical margins may benefit from aggressive adjuvant local therapy as they are at risk for harboring small-volume residual disease not seen on histopathologic evaluation.

New technological advancements in radiation therapy, such as those utilized in SABR, allow delivery of high-dose oligofractionated treatments, leading to reduction in time and inconvenience for patients. These techniques have been applied to locally advanced, unresectable PA, but not as adjunctive treatment in surgically resectable disease [22-24]. Previous experience with SABR in PA has exposed some of the limitations when performing high dose per fraction treatment near radiosensitive normal structures such as the duodenum. Yet, if an area at high risk of true local failure (the retroperitoneal margin space) were targeted instead of the entire tumor burden, these normal tissues could be spared to a greater extent than in these prior experiences. In addition, if SABR techniques are used, radiation can be delivered in a single fraction (as in our study), or a few fractions, leading to reduction of treatment time and the delay in time to receiving chemotherapy in these patients. These hypotheses have driven exploration of neoadjuvant ablative SABR in a single fraction focusing on targeting the retroperitoneal margin where local failure is common. MIT, as we have termed it, is being evaluated in a phase I trial at our institution, where patients with surgically resectable PA receive 30 Gy in 1 fraction to the sub-GTV volume targeting the at-risk margin (not the tumor) prior to proceeding to pancreatoduodenectomy. This dose has been previously applied in intraoperative irradiation studies for essentially the same reason, although to a larger target space than the sub-GTV in our study [33]. Patients will receive adjuvant systemic chemotherapy after surgical resection as is normally practiced in the U.S. We will evaluate preoperative acute toxicity related to SABR delivery as well as post-operative mortality in hopes of characterizing the associated toxicities of this novel idea. We will also characterize secondary endpoints such as surgical margin positivity and local control rates.

The first aspect of our study evaluated motion of regional structures around the sub-GTV to characterize appropriate margins. Because the sub-GTV represents an area along the retroperitoneal margin and is not a discrete structure such as the tumor volume itself (as in SABR for lung cancer), motion of this margin must be determined by the motion of adjacent structures on which the margin is based. For the sub-GTV volume in this case, the SMA itself most likely represents the motion associated with the sub-GTV volume. We have hypothesized that motion of the SMA would be small, but there are no detailed descriptions within the current literature evaluating this motion. As expected, mean motion of the SMA was small in any one direction (< 3 mm) (Table 1), and was not significantly affected by AC. Yet, vector motion of the SMA was significantly reduced by AC in our study of 10 patients. Because the motion of the SMA is not evaluable prior to simulation utilizing fluoroscopy, the use of AC within the trial is based on pre-simulation fluoroscopic evaluation of fiducial motion. Fiducials are placed in the head of the pancreas prior to simulation and motion of these fiducials has been > 5 mm on fluoroscopy without compression in all four patients enrolled on the trial thus far. AC has been used in these patients to reduce this motion, but the effect of AC on SMA motion was not known until this study was initiated. Other regional respiratory-associated organ motion has been well characterized within the literature utilizing several types of methods including ultrasound, fluoroscopy, or 4DCT [34-39]. In addition, the effect of abdominal compression on organ motion has also been characterized [25,36]. Smaller overall organ motion seen in our study may be due to the methods used in both breathing instruction and evaluation of movement. Our study did not include an audio respiratory coach such as was used in the Brandner et al. study [40]. An audio coach instructing the patient when to inspire, may actually cause an increase in respiratory amplitude and thus greater respiratory movement than without a coach [41]. Audio and audiovisual coaching has been shown to make the respiratory cycle more reproducible, but may be one reason for slightly increased respiratory associated organ motion in studies using this method [41]. In general, the relatively low amplitude organ motion seen with free breathing in this region was reduced, although not significantly with the use of AC. In addition, there may be a difference in motion of surrounding organs due to the presence of a pancreatic head tumor and associated mass effect from the desmoplastic reaction often associated with these tumors. Some of the patients analyzed for motion did not have pancreatic tumors, and thus may have different motion characteristics. After further analyzation, however, we found no significant difference in the organ motion amplitude for the patients with or without pancreatic tumors. The tumors treated with the radiation approach described in this manuscript are resectable and thus in general likely to be low-volume tumors. By definition, they are not attached directly to any surrounding organs other than the duodenum. Because of limitations of this study including patient number, single time point 4DCT acquisition, and inherent errors related to 4DCT acquisition and reconstruction,[41] we will continue to evaluate motion on a individual patient basis to determine the appropriate use of AC in this setting.

In addition to evaluating the motion effects of AC, it is clear that the AC plate itself displaces abdominal organs causing displacements and deformations. There was some concern that displacement of structures such as the stomach and transverse colon towards the sub-GTV target could increase dose to these structures. Thus, identical plans were performed on planning CT scans both with and without abdominal compression to see the effect of the compression plate on OAR dose. As shown in Table 2, no significant changes were seen in mean dose to any of the regional OARs including the stomach and traverse colon. We also did analysis on minimum, maximum, and dose to various volumes of OARs (not shown), which also did not change significantly with the application of abdominal compression. Again, the presence or absence of a pancreatic tumor could affect the effect of AC on surrounding organs in this heterogeneous patient population. Yet, on further analysis, no dosimetric difference was seen when the patients were separated into those with or without pancreatic tumors. Based on this data, we are reassured that AC can be applied safely to control motion in SABR of the pancreas without potentially increasing dose significantly to regional OARs of interest.

Finally, because SABR to this region had not been performed at our institution prior to initiation of this study, little was known about the best planning technique for delivery of single fraction ablative radiotherapy to the planned volume of interest. Thus, we compared doses to regional OARs for three different planning methods including non-coplanar 3DSABR, coplanar IMRT, and CK. Various significant results for OAR dose were highlighted in the results section. In general, non-coplanar strategies like 3DSABR and CK were able to reduce dose to vessels or structures in axial plane of the PTV such as the renal vessels and kidneys because these techniques enable a more isotropic dose falloff, especially in the axial plane [26]. It is important to state that objectives for the kidneys and renal vessels were nonetheless met in all plans such that toxicity is unlikely to be affected by observed differences. 3DSABR and CK had higher doses to vessels immediately adjacent to the PTV including the celiac trunk and SMA. This difference is likely associated with the prescription method of each planning technique. 3DSABR and CK were typically prescribed to the 60-80% isodose line, while IMRT was prescribed to 95-100% of the PTV maximum dose. Often, a portion of the SMA and celiac trunk are included in the PTV volume after expansion. Thus, hot spots within the PTV itself can affect maximum doses to the SMA and celiac trunk. The tolerance of vessels such as the SMA to external beam irradiation is informed in part by experience from intraoperative irradiation studies [42]. Radiation tolerance of the superior mesenteric plexus is less well appreciated. Patients will be monitored long term for such rarely reported toxicities as infarction from vessel atherosclerosis or aneurysm development, as well as autonomic neural effects. IMRT was also able to improve upon dose to the liver and transverse colon, organs at further distance from the target volume than those discussed thus far. Again, specific constraints for single fraction SABR were met for these structures for the other planning methods, and thus no conclusions on correlation to toxicity can be made. Finally, CK and IMRT both improved CI over 3DSABR. The PTV volume described has a concave portion, limiting 3DSABR's ability to achieve a high conformality index, unlike typical tumors, which are spherical. Thus, the ability of CK and IMRT to shape dose around concavities lead to significantly better CI in these ten patients. From our limited study, no superior planning method was determined. Because of increased conformality and reduced dose to adjacent vessels, IMRT planning technique is utilized at our institution thus far for treatment on this clinical trial.

## Conclusions

In conclusion, this study introduces a new treatment concept, coined MIT, to preoperatively treat the retroperitoneal margin in resectable PA to a single high dose of radiation and is being evaluated in a phase I trial at our institution. In preparation for opening this trial, we have examined several novel aspects related to radiation planning techniques including regional respiratory-associated motion that may affect target coverage and planning margins and the effect of AC on this motion. In addition, we have determined that AC does not affect the dose to regional OARs around our PTV. Finally, we have evaluated three different planning techniques to deliver single fraction SABR to this region, and described strengths and weaknesses of each technique.

## Competing interests

The authors declare that they have no competing interests.

## Authors' contributions

JH carried out patient identification, study location, and participated in contouring and planning and statistical analysis and served as the primary author of the manuscript text. RB participated in contouring and planning and the was secondary author of the manuscript text. JCM participated in patient selection and volume definition and serves as the primary surgical oncologist and primary investigator on the clinical trial. RS participated in patient selection and volume definition and serves as a coinvestigator on the clinical trial. ER participated in dosimetric planning for IMRT and 3DSABR. CD participated in dosimetric planning for CK. RA participated in patient selection and protocol idea creation and serves as a co-investigator on the clinical trial. TB participated in creating the study idea. TS participated in creating the study idea, monitoring the study progress, and editing the manuscript. RT participated in creating the study idea, monitoring the study progress, editing the manuscript, and serves as a co-investigator on the clincal trial JJM participated as a primary investigator of the clinical trial, creating the study idea, monitoring the study progress, and editing the manuscript. All authors read and approved the final manuscript.
